# Epstein-Barr Virus Specific Antibody Response in Multiple Sclerosis Patients during 21 Months of Natalizumab Treatment

**DOI:** 10.1155/2015/901312

**Published:** 2015-05-26

**Authors:** Massimiliano Castellazzi, Serena Delbue, Francesca Elia, Matteo Gastaldi, Diego Franciotta, Roberta Rizzo, Tiziana Bellini, Roberto Bergamaschi, Enrico Granieri, Enrico Fainardi

**Affiliations:** ^1^Department of Biomedical and Specialist Surgical Sciences, University of Ferrara, 44124 Ferrara, Italy; ^2^Department of Biomedical, Surgical and Dental Sciences, University of Milan, 20122 Milan, Italy; ^3^Department of General Neurology, National Neurological Institute C. Mondino, 27100 Pavia, Italy; ^4^Department of Medical Sciences, University of Ferrara, 44121 Ferrara, Italy; ^5^Department of Neurosciences and Rehabilitation, Azienda Ospedaliero-Universitaria, 44124 Ferrara, Italy

## Abstract

Multiple sclerosis (MS) is a chronic inflammatory autoimmune disease of the central nervous system. Natalizumab, a humanized anti-*α*4 integrin monoclonal antibody, is a highly effective treatment approved for MS. An association between MS and an exposure to Epstein-Barr Virus (EBV) sustained by the levels of antiviral capsid antigen (VCA) and anti-Epstein-Barr nuclear antigen-1 (EBNA-1) IgG has been described. Our goal was to verify the utility of EBV-specific IgG as a marker in Natalizumab treated MS. Twenty patients (17 female and 3 male) in treatment with Natalizumab were enrolled. Serum levels of anti-VCA and anti-EBNA-1 IgG were determined and expressed as arbitrary units (AU) before treatment and every three months for 21 months of therapy. Anti-VCA IgG levels were increased at the 15th month (235410 ± 196712 AU) comparing with the 3rd (98146 ± 47145 AU) and the 6th (109866 ± 52270 AU) months of therapy (*p* < 0.05). No significant differences were found for serum anti-EBNA-1 IgG levels. Our data indicate that a transient, self-limited, EBV reactivation can occur in MS during Natalizumab therapy but our results do not support the use of serum EBV-specific antibody levels as biomarkers for monitoring therapeutic response to Natalizumab in the course of MS.

## 1. Introduction

Multiple sclerosis (MS) is considered an autoimmune chronic inflammatory disease of the central nervous system (CNS) of unclear etiology that is marked by demyelination and axonal loss [1]. MS usually occurs in young adults, is more frequent in women than in men, and is characterized by clinical attacks or exacerbations, called relapses, which typically show a dissemination in space and time [2]. Lymphocyte migration across the blood-brain barrier (BBB) is thought to be a crucial step in the initiation and maintenance of brain inflammatory reaction [3]. The interaction of *α*4*β*1 integrin, a protein on the surface of lymphocytes, with vascular-cell adhesion molecule-1 (VCAM-1), which is expressed on the surface of vascular endothelial cells in brain and spinal cord blood vessels, mediates the adhesion and migration of lymphocytes in areas of CNS inflammation [4]. Natalizumab (Tysabri, Biogen Idec Inc., Cambridge, Massachusetts, USA), a humanized anti-*α*4 integrin monoclonal antibody, is a highly effective treatment approved for relapsing remitting multiple sclerosis (RRMS) [5, 6]. Natalizumab is administered intravenously to RRMS patients once every 4 weeks in a dose of 300 mg, and its efficacy in substantially reducing relapse rate and the progression of disability has been shown in clinical trials [7]. Therefore, Natalizumab is currently used as second-line treatment in MS patients who have a suboptimal response to first-line disease-modifying therapies or as first-line therapy in those with highly active disease [8]. However, despite the undisputable benefits, anti-*α*4 integrin treatment is associated with John Cunningham Virus- (JCV-) mediated progressive multifocal leukoencephalopathy (PML), an unfavourable and severe adverse event [9]. Although disease etiology remains largely unknown, epidemiological studies suggest that the combination of exposure to an environmental factor, such as an infectious agent, and genetic predisposition could play a crucial role in MS pathogenesis [10]. In this setting, an ideal candidate is represented by Epstein-Barr Virus (EBV), a human *γ*-herpesvirus which can infect, activate, and latently persist in B-lymphocytes for life [11]. In a meta-analysis of previously published case-control observational studies [12], the authors found an association between MS and exposure to EBV which was particularly sustained by the levels of antiviral capsid antigen (VCA) IgG and anti-Epstein-Barr nuclear antigen-1 (EBNA-1) IgG. No significant association was found when studying anti-early antigen (EA) IgG. In addition, it has been demonstrated that a past infectious mononucleosis (IM) was frequent and the seroprevalence of anti-EBNA-1 and anti-VCA IgG was higher in MS patients than in controls [13, 14]. Elevated serum levels of anti-EBNA-1 IgG were associated with an increased risk of developing MS [15] and disease activity [16] and were found to be predicting factors for the conversion from clinical isolated syndrome (CIS) to definite MS [17]. On the other hand, high serum concentrations of anti-VCA IgG were related to grey matter atrophy [18]. Taken together, these observations suggest that EBV-specific antibody response could be used as a marker for disease development and activity. This possibility was further corroborated by the repeated evidence that anti-EBNA-1 serum titers were greater in MS than in controls [19, 20]. Nevertheless, whether serum concentrations of anti-EBV antibodies can actually serve as biomarker for monitoring MS treatment response is still to be established. For this reason, the potential of serum concentrations of anti-EBNA-1 as indicators of MS disease activity was recently tested in a small population of treatment-naïve MS patients before and after 12 months of therapy with Natalizumab, with negative results [21]. Therefore, the aim of our study was to verify the effective utility of EBV-specific serum antibodies as a marker for the response to treatment with Natalizumab in a cohort of relapsing remitting multiple sclerosis (RRMS) patients during 21 months of therapy.

## 2. Materials and Methods

### 2.1. Study Design and Sample Handling

This study included 20 consecutive patients (17 female and 3 male) with definite RRMS [22] in treatment with Natalizumab after discontinuation of therapy with immunomodulatory or immunosuppressive drugs (6 on glatiramer acetate, 5 on interferon *β*-1a, 4 on interferon *β*-1b, 4 on mitoxantrone, and 1 on cyclophosphamide) due to unresponsiveness represented by the occurrence of at least one relapse in the previous year. Patients were enrolled at the “Fondazione Istituto Neurologico C. Mondino” in Pavia. Serum samples were collected at baseline and consecutively at 3, 6, 9, 12, 15, 18, and 21 months after the initiation of Natalizumab therapy. At all time points, (a) disease severity was scored using Kurtzke's Expanded Disability Status Scale (EDSS) [23]; (b) presence of relapse, defined as the onset of new or recurrent symptoms or signs or the worsening of already present neurological abnormalities persisting for at least 24 h in the absence of fever and preceded by at least 1 month of stable or improved neurological state, was recorded as clinical activity; and (c) anti-JCV serostatus was determined to prevent the risk of PML in accordance with previous validated enzyme-linked immunosorbent assay (ELISA) protocol [24]. Disability progression during Natalizumab treatment was defined as an increase of one point on EDSS score from baseline [5]. All patients underwent brain Magnetic Resonance Imaging (MRI) scans at entry and at the end of the study and the occurrence of a new lesion on T2-weighted scans and/or a new gadolinium- (Gd-) enhancing lesion on T1-weighted scans was defined as MRI activity [22]. None of the patients had been receiving corticosteroids at the time of sample collection. The approval of the Committee for Medical Ethics in Research as well as written informed consent from all subjects participating in the study was obtained for experiments involving human subjects.

### 2.2. Serum Levels of Anti-Epstein-Barr Virus Antibodies

Serum concentrations of anti-EBNA-1 and anti-VCA IgG were measured by ELISA using commercially available ELISA kits (Novagnost EBV-EBNA1 IgG and EBV-VCA IgG, codes numbers EBVG0580DB and EBVG0150DB, resp.) as described elsewhere [19, 20]. All reagents, plates, and peroxidase-conjugated antibody were included in the kits. Microtiter strip wells were precoated with recombinant EBNA-1 and synthetic VCA (p18) antigens, respectively. A reference curve was generated in each assay using six serial dilutions of pooled EBV-high-positive serum samples ranging between 0.1 and 2.0 OD. Serum samples, prediluted 1 : 100 or 1 : 1200, were dispensed in duplicate into two microtiter plates, one precoated with EBNA-1 and the other precoated with VCA. A reference curve was generated in each assay using the pooled serum samples by plotting the concentrations, expressed as arbitrary units (AU), versus the relative optical density (OD) values. For each sample, anti-EBNA-1 and anti-VCA IgG concentrations were obtained by multiplying AU value for the corresponding dilution factor.

### 2.3. Data Analysis

Statistical analysis was performed with GraphPad Prism. After checking data for normality by means of the Kolmogorov-Smirnov test, a normality of data distribution was rejected in several variables. Therefore, statistical analysis was performed through a nonparametric approach. More precisely, continuous variables were compared using Kruskal-Wallis test and Friedman test for repeated measures. Dunn's test correction was utilized for multiple comparisons. A value of *p* < 0.05 was accepted as statistically significant.

## 3. Results

**Figure 1:**
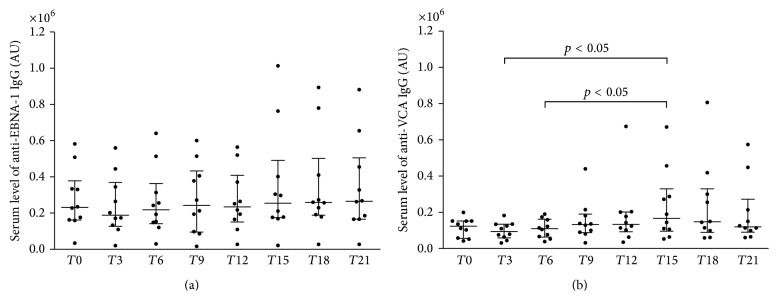
Longitudinal fluctuations of anti-EBNA-1 and anti-VCA IgG in the ten patients with relapsing remitting multiple sclerosis (RRMS) treated with Natalizumab for 21 months in which blood samples were taken at every time point. Serum levels of anti-EBNA-1 and anti-VCA IgG were different among various time points (Friedman test: *p* < 0.05 and *p* < 0.01, resp.). Serum levels of anti-VCA IgG were more elevated at *T*15 compared to *T*3 and *T*6 (Dunn's posttest: *p* < 0.05) whereas no differences were found comparing each time point for EBNA-1 IgG levels in a post hoc analysis. AU = arbitrary units; EBNA-1 = Epstein-Barr nuclear antigen-1; *T*0 = baseline; *T*3 = the 3rd month; *T*6 = the 6th month; *T*9 = the 9th month; *T*12 = the 12th month; *T*15 = the 15th month; *T*18 = the 18th month; *T*21 = the 21st month; VCA = viral capsid antigen. Horizontal bars indicate medians and error bars correspond to interquartile range.

Demographic and clinical characteristics of 20 RRMS patients receiving Natalizumab are listed in Table 1. During Natalizumab treatment, (a) five patients had relapses (3 patients had 1 relapse between baseline and 3 months, one had 2 relapses between 6 and 9 months and at 12 months, and one had 2 relapses between 9 and 12 months and between 18 and 21 months); (b) no patients had a progression of disability from baseline; and (c) four patients showed a new *T*2 and/or Gd-enhancing lesions on the last MRI examination at 21 months, but none of these had relapses. No patients showed anti-JCV seropositive during the 21 months of Natalizumab therapy. Only for ten patients, it was possible to perform a longitudinal determination of EBV-specific antibodies at each time point (of these, 2 patients had 1 relapse between baseline and 3 months and one had 2 relapses between 9 and 12 months and between 18 and 21 months), whereas for the remaining ten patients the timing of sample collection was not sequential and so resulted incomplete. Serum levels of anti-EBNA-1 and anti-VCA IgG were detected in all samples. As reported in Table 2, when MS patients were analyzed as a whole, no significant differences were found for serum concentrations of either anti-EBNA-1 or anti-VCA IgG levels among the various time points. Conversely, when we evaluated only MS patients in whom serum samples were available at all time points (Figure 1), serum levels of anti-EBNA-1 and anti-VCA IgG were statistically different among the various time points (Friedman test: *p* < 0.05 and *p* < 0.01, resp.). However, post hoc analysis revealed that while anti-VCA IgG levels were significantly higher at the 15th month than at the 3rd and the 6th months after the beginning of therapy (Dunn's posttest: *p* < 0.05), no significant differences were found for serum anti-EBNA-1 IgG levels among the different time points.

## 4. Discussion

This study has demonstrated for the first time that temporal fluctuations of serum levels of EBV-specific IgG in RRMS could be affected by treatment with Natalizumab. In recent decades, several studies have shown that an association can exist between antibodies specific for EBV antigens, in particular EBNA-1 and VCA, and some clinical features of MS, such as disease initiation and activity [11–18]. Thus, these antibodies are considered as putative biomarkers which may be useful for describing the natural history of the disease or “type 0 biomarkers” following the definition of Bielekova and Martin [25]. The purpose of our study was to investigate whether EBV-specific antibodies could also be used in RRMS patients as “type I biomarkers” to capture the effects of Natalizumab intervention in accordance with its mechanism of action [25]. In agreement with other investigators [21], our results confirmed that anti-EBV antibodies are not a useful marker of disease activity in patients treated with Natalizumab. In fact, anti-VCA IgG serum levels peaked at the 15th month after the start of therapy when no patients had clinical activity, as indicated by lack of the occurrence of a relapse. In addition, MRI activity was present in only four patients on the last examination at the 21st month when serum concentrations of EBV-specific antibodies did not differ compared to baseline and the other time points. However, here we documented that serum levels of anti-VCA IgG were transiently increased during Natalizumab therapy since they were more elevated at the 15th month than at the 3rd and the 6th months of treatment. This finding is difficult to interpret in the absence of clinical evidence of disease activity. The presence of a dysregulated EBV infection of the CNS has recently been suggested [26]. Therefore, we are tempted to speculate that Natalizumab treatment, interfering with the EBV-specific CD8+ trafficking into CNS, could promote an EBV reactivation within the brain with a consequent release of antigens from the CNS to the periphery. Thus, the presence of these antigens may induce a peripheral production of EBV-specific antibodies. Nevertheless, the lacking of a simultaneous JCV reactivation, as shown by JCV-specific seronegativity at the same time point, weakens the consistency of this hypothesis [21]. Alternatively, this transient elevation of anti-VCA IgG may represent a reactivation of EBV infection in systemic compartment due to a prolonged immunosuppression in peripheral organs induced by Natalizumab [27]. However, this possibility is not sustained by the demonstration that the amounts of blood activated CD8+ T-cells releasing proinflammatory cytokines were enhanced in MS patients treated with Natalizumab [28]. Moreover, this speculation could be confirmed by the concomitant increased of anti-early antigen (EA) antibodies which, however, have not been measured in this study due to the conflicting results previously obtained on their ability in identifying EBV reactivation [12]. On the other hand, it is interesting to note that only anti-VCA IgG and not anti-EBNA-1 IgG was increased. As VCA are viral surface proteins and EBNA-1 represent nuclear viral proteins, this result could be explained by the different biological significance of these two different antibodies. In fact, it has been postulated that EBV acts as an intermittently cytopathic virus [29] that latently persists life-long in B-cells and causes recurring reactivations [30]. Lytic proteins, including VCA, are expressed during replication whereas latent genes, including EBNAs, are expressed in the growth phases of infection [11]. In this way, the transient increase in the serum anti-VCA IgG levels we observed at the 15th month may reflect an ongoing and self-limiting replicative EBV infection [29, 30]. The main limitations of this study were certainly the small sample size and the presence of some patients in whom not all serum samples were available. Another limiting factor is the lack of samples collected at the time of relapse which could contribute to a more reliable identification of possible correlations between EBV-specific antibodies and disease activity. Taken together, although these data indicate that a transient self-limited EBV reactivation can occur in RRMS during Natalizumab therapy, they argue against the use of serum EBV-specific antibody levels as biomarkers for monitoring therapeutic response to Natalizumab in the course of RRMS. However, future studies are needed to verify the actual significance of anti-EBV antibodies in MS patients who are undergoing Natalizumab therapy.

## Figures and Tables

**Table 1:** Demographic, clinical, and radiological characteristics in 20 relapsing remitting multiple sclerosis (RRMS) patients receiving Natalizumab.

Sex: female/male	17/3
Age at entry, years: mean ± SD	34.2 ± 9.8
Disease duration at baseline, years (mean ± SD)	10.2 ± 6.2
Disease severity at baseline, EDSS: mean ± SD	1.3 ± 1.5
EDSS after 21 months of treatment (mean ± SD)	1.7 ± 1.7
Relapsing/nonrelapsing patients during 21 months of therapy	5/15
Patients with/without new MRI lesions at the end of treatment	4/20

EDSS = Expanded Disability Status Scale; MRI = Magnetic Resonance Imaging; SD = standard deviation.

**Table 2:** Longitudinal fluctuations in serum anti-EBNA-1 and anti-VCA IgG levels in relapsing remitting multiple sclerosis (RRMS) patients, considered as a whole, during 21 months of Natalizumab treatment.

Time point (sample)	Serum anti-EBNA-1 IgG levels (AU) median, IQR	Serum anti-VCA IgG levels (AU) median, IQR
*T*0 (*n* = 16)	273750, 160073–411955	107720, 44682–151729
*T*3 (*n* = 19)	264992, 133546–372122	82629, 45782–134306
*T*6 (*n* = 19)	299852, 149020–415994	103548, 52914–158579
*T*9 (*n* = 19)	272304, 151911–433509	100277, 73889–172901
*T*12 (*n* = 20)	234446, 159224–369656	112400, 59598–195399
*T*15 (*n* = 18)	218906, 164613–356031	110428, 88676–276036
*T*18 (*n* = 19)	230182, 136390–410000	145650, 98579–310823
*T*21 (*n* = 18)	239703, 163733–366165	115988, 77566–379377

AU = arbitrary units; EBNA-1 = Epstein-Barr nuclear antigen-1; IQR = interquartile range; SD = standard deviation; *T*0 = baseline; *T*3 = the 3rd month; *T*6 = the 6th month; *T*9 = the 9th month; *T*12 = the 12th month; *T*15 = the 15th month; *T*18 = the 18th month; *T*21 = the 21st month; VCA = Epstein-Barr viral capsid antigen.
